# miR-128 Regulates Tumor Cell CD47 Expression and Promotes Anti-tumor Immunity in Pancreatic Cancer

**DOI:** 10.3389/fimmu.2020.00890

**Published:** 2020-05-27

**Authors:** Qing Xi, Ying Chen, Guang-Ze Yang, Jie-You Zhang, Li-Juan Zhang, Xiang-Dong Guo, Jing-Yi Zhao, Zhen-Yi Xue, Yan Li, Rongxin Zhang

**Affiliations:** ^1^Department of Immunology, Key Laboratory of Immune Microenvironment and Diseases of Educational Ministry of China, Tianjin Key Laboratory of Cellular and Molecular Immunology, Tianjin Medical University, Tianjin, China; ^2^Guangdong Province Key Laboratory for Biotechnology Drug Candidates, Institute of Basic Medical Sciences and Department of Biotechnology, School of Life Sciences and Biopharmaceutics, Guangdong Pharmaceutical University, Guangzhou, China

**Keywords:** miR-128, pancreatic adenocarcinoma (PDAC), zinc finger E-box-binding homeobox 1 (ZEB1), cluster of differentiation 47 (CD47), tumor immunity

## Abstract

Pancreatic adenocarcinoma (PDAC) is a highly fatal disease worldwide. MicroRNAs (miRNAs) could regulate the protein-coding RNAs related to tumor growth, invasion, and immune evasion. Therefore, the investigation of novel miRNAs may be helpful in the development of more effective therapies for PDAC. In this study, we investigated the role and mechanism of action of miR-128 in PDAC. By using bioinformatics methods, we found that decreased expression of miR-128 was associated with poor overall survival of PDAC. miR-128 was inversely correlated with cluster of differentiation 47 (CD47), which was positively related to zinc finger E-box-binding homeobox 1 (ZEB1) in PDAC. Through *in vivo* experiments, we found that miR-128 could suppress the growth and metastasis of PDAC. Analysis of the immune microenvironment demonstrated that overexpression of miR-128 on tumor cells could increase the percentages of dendritic cells (DCs), CD8^+^ T lymphocytes, and natural killer T cells (NKT) in the tumor and spleen, consequently enhancing anti-tumor immunity. *In vitro* assays showed that miR-128 could inhibit cell proliferation, clonogenicity, migration, and invasion in Panc02 cells and could also enhance the phagocytosis of macrophages and the activity of DCs. Western blot and qRT-PCR confirmed that miR-128 could regulate ZEB1 and further inhibit CD47 in pancreatic cancer cells. Therefore, we identified a novel regulatory anti-tumor mechanism by miR-128 in PDAC, which may serve as a novel therapy for PDAC.

## Introduction

Pancreatic adenocarcinoma (PDAC) is one of the most aggressive and lethal solid tumors and is currently the fourth most common cause of cancer-related mortality in the developed world (1). The 5-year survival rate is only at 8% (2). It is predicted to become the second leading cause of cancer-related death within a decade if its trend continues (3). Therefore, studies are urgently needed to determine the mechanism of pancreatic carcinogenesis and progression to identify new therapeutic targets for treating patients with PDAC. The concepts of precision oncology and PDAC subtypes include genetic and transcriptional changes in epithelial cells, as well as complex cellular changes in the tumor microenvironment (TME) and multiple biochemical or epigenetic crosstalks in the tumor matrix of PDAC. The immune-checkpoint-based cancer immunotherapy revolution has transformed the field of immuno-oncology. Antibodies against inhibitory receptors such as programmed death (PD)-1/PD-L1 have shown significant efficacy against several cancers (4). However, many patients fail to respond to current immunotherapies or have short-lived responses, so it is important to identify other forms of immunotherapy to improve the cure rate of patients. Cluster of differentiation 47 (CD47) (also known as integrin-associated protein) is a cell surface transmembrane protein of the immunoglobulin superfamily that plays important roles in self-recognition (5). It is ubiquitously expressed on all normal cells and is often overexpressed on various solid and hematologic cancer cells; its overexpression is clinically correlated with poor prognoses (6, 7). Blocking CD47 could shrink pancreatic cancers (8). CD47 is a checkpoint molecule for both innate and adaptive immunity for tumor immune evasion and is thus a promising target for cancer immunotherapy (9, 10). Through interacting with its ligands signal regulatory protein α (SIRPα) on phagocytes, CD47 transmits a “don't eat me” signal that prevents cell engulfment (11). It has become increasingly appreciated that the therapeutic efficacy of CD47 blockade in tumor models requires DCs to activate adaptive immune response (12). Epithelial–mesenchymal transition (EMT) is a process in which epithelial cells acquire mesenchymal features and is associated with tumor initiation, malignant progression, tumor cell migration, invasion and metastatic spread, tumor stemness, and resistance to therapy (13). It is orchestrated by a series of master EMT-inducing transcription factors (EMT-TFs) and often defined by the loss of the epithelial marker E-cadherin and the gain of the mesenchymal marker Vimentin (14). The zinc finger E-box-binding homeobox 1 (ZEB1) is a crucial EMT activator in several cancers (15, 16), closely related to tumor initiation and malignant progression (13, 17), which can also regulate CD47 (18).

MicroRNAs (miRNAs) are ~22 nt in length, could repress target genes by directly binding to the 3′untranslated regions (3′UTRs) and play critical roles in tumorigenesis, development, and prognosis (19). Studies have reported that several miRNAs could inhibit tumor growth by regulating the expression of CD47 in cancer cells (20, 21). miR-128 is a kind of intronic miRNA and is encoded by two distinct genes, miR-128-1 and miR-128-2, which are embedded in the introns of the R3HDM1 (R3H domain containing 1) and RCS (ARPP-21, cyclic AMP-regulated phosphoprotein) genes, respectively (22). Both miR-128-1 and miR-128-2 are processed to generate the same mature miRNAs with identical sequences (23), namely, miR-128. miR-128 is associated with various carcinomas such as ovarian cancer (24), colorectal cancer (25), and breast cancer (26). However, little is known about the role of miR-128 in PDAC. In this study, we uncovered a novel mechanism of promotion of anti-tumor immunity by miR-128 through regulating the ZEB1/CD47 axis, thus linking an EMT regulatory program to anti-tumor immunity in PDAC.

## Materials and Methods

### Cell Culture

Mouse PDAC cells Panc02 were established by Corbett et al. (27). The mouse PDAC cell line H7 and human PDAC cell line PANC1 were originally obtained from the American Type Culture Collection. The cells were cultured in DMEM medium (Gibco, USA) containing 10% fetal bovine serum (FBS; Hyclone, USA), 100 U/ml of penicillin, and 100 μg/ml of streptomycin, incubated in a humidified atmosphere at 37°C with 5% CO_2_. Passaging was performed at 80% confluence with 0.25% trypsin.

### siRNA Transfection

The miR-128 mimic, control mimic, ZEB1 siRNA, and control siRNA were purchased from RiboBio (Guangzhou, China). The cells were treated with mimics using Lipofectamine RNAiMAX (Invitrogen, USA) according to the manufacturer's instructions. Briefly, both miRNA (final concentration, 25 nM) and Lipofectamine RNAiMAX reagent were diluted in Opti-MEM medium. An equivalent volume of Lipofectamine RNAiMAX reagent (1:1 ratio) was added into miRNA solution and then incubated for 5 min at room temperature. The mixture was added to 2.5 × 10^5^ cells/well in six-well culture plates. The transfected cells were incubated in a humidified environment at 37°C and 5% CO_2_ for 24 and 48 h.

### Lentiviral Constructs and Infection

miR-128 overexpression lentivirus vector pGLV3–H1–miR-128–GFP–Puro (GenePharma, Shanghai, China) or pGLV3–H1–GFP–Puro empty lentiviral vector (miR-NC) was transfected into HEK293T cells with the packaging vectors pMD2.G (Addgene) and psPAX2 (Addgene) using PEI (Polyscience). After 48 and 72 h, the culture medium was collected and centrifuged at 2,000 × g for 5 min to remove cell debris. The supernatant was filtered with a microfiltration membrane and subsequently placed in 40-ml ultracentrifugation tubes; 1/4 volume PEG 8000 was added into the supernatant and then incubated overnight at 4°C with shaking. The next day, the samples were centrifuged at 4,000 rpm/min for 30 min at 4°C and resuspended for virus precipitation to collect pGLV3–H1–miR−128–GFP–Puro virus. The negative control lentivirus was obtained similarly. The Panc02 cells were infected with the lentivirus collected above and selected by puromycin.

### Quantitative Real-Time PCR (qRT-PCR)

The total RNA was extracted using TRIzol reagent (Invitrogen, USA). A quantity of 2 μg of total RNA was converted into cDNA with M-MLV reverse transcriptase (Invitrogen, USA). The qRT-PCR was performed using SYBR Green mix (DBI Bioscience, Germany) in an ABI PRISM 7500 Fast Real Time PCR System (Applied Biosystems, USA). The fold changes were calculated by 2^−ΔΔCt^. The primers for miR-128 were purchased from RiboBio (Guangzhou, China). Other primers were synthesized by Sangon Biotech (Shanghai, China) and are listed below: ZEB1 (Forward: 5′-GCTGGCAAGACAACGTGAAAG-3′; Reverse: 5′-GCCTCAGGATAAATGACGGC-3′). CD47 (Forward: 5′-TGCGGTTCAGCTCAACTACTG-3′; Reverse: 5′-GCTTTGCGCCTCCACATTAC-3′). GAPDH (Forward: 5′-CCATGTTTGTGATGGGTGTGAACCA-3′; Reverse: 5′-ACCAGTGGATGCAGGGATGATGTTC-3′).

### Western Blot Analysis

The whole cell lysates were prepared using RIPA lysis buffer in the presence of 1% phosphatase inhibitor cocktail and 1 mM phenylmethanesulfonyl fluoride (PMSF). The protein was subjected to SDS-PAGE after boiling for 10 min in 1 × SDS loading buffer and electrophoresed at 80 V for 30 min followed by 120 V for 1 h, then transferred to a PVDF membrane (Millipore, USA) at 180 mA for 2 h. After blocking with 5% non-fat milk at room temperature for 1 h, the membranes were incubated with the primary antibodies overnight at 4°C. The anti-GAPDH antibody was purchased from Sungene (China; 1:1,000). The anti-ZEB1 antibody was purchased from Cell Signaling Technology (CST; 1:1,000). The anti-CD47 antibody was purchased from ABclonal (China; 1:1,000). After incubating with horseradish peroxidase-conjugated secondary antibody (CST, 1:2,000), immunoreactive bands were visualized using the ECL Western Blotting Detection System (Millipore, USA).

### Cell Proliferation Assays

For the analysis of proliferation, the Panc02 control and Panc02 miR-128 overexpression cell lines were seeded in 96-well-culture plates (2,000 cells per well) and incubated for an appropriate time. Cell proliferation was assessed using a Cell Counting Kit-8 (Dojindo, Kumamoto, Japan) as follows. Ten microliters of CCK-8 solution was added to the medium at the appropriate time, and cells were incubated for 4 h in a humidified atmosphere with 5% CO_2_; the amount of orange formazan staining was calculated by measuring the absorbance at 450 nm using a microplate reader (BioTek Instruments, USA).

### Clonogenicity Assays

The Panc02 control and Panc02 miR-128 stable overexpression cell lines were seeded into six-well-culture plates (1,000/well) and cultured in complete DMEM. The cells were then incubated for ~2 weeks at 37°C in a 5% CO_2_ incubator. The colonies were stained with crystal violet and quantitated.

### Wound Healing Assay

The Panc02 control and Panc02 miR-128 overexpression cell lines were seeded into six-well-culture plates and cultured in complete DMEM. After reaching ~70% confluence, the medium was replaced with serum-free medium. Following overnight incubation, the cells reached at least 95% confluence, forming a confluent monolayer. Linear scratches were made using a 10-μl micropipette tip. The cells were further cultured in DMEM medium containing 2% FBS. The wound width was photographed using an optical microscope (Olympus, Japan) at 0 and 24 h. To evaluate wound closure, three randomly selected points along each wound were marked. Each experiment was performed in triplicate. The measurements were obtained by measuring the distance between the wound edges using Image J software.

### Transwell Cell Invasion Assays

For cell invasion, polyethylene terephthalate (PET) cell culture inserts with 8.0-μm pores (BD Biosciences, Franklin lakes, NJ, USA) were placed in a 24-well-culture plate and equilibrated for 30 min; the upper chamber was placed with Matrigel (BD Biosciences). Six hundred microliters of culture medium and 20% FBS were added into the lower chamber. Cells were harvested, washed with PBS three times, and resuspended in serum-free medium at 5 × 10^5^ cells/ml. The cells (100 μl) were added to the upper chamber and incubated for 24 h at 37°C with 5% CO_2_.

### Animals and Tumor Formation Study

C57BL/6 mice were purchased from the Academy of Military Medical Science (Beijing, China). All mice used in this study were 6–8 weeks old and housed in microisolator cages in a specific pathogen-free animal facility at the Experimental Animal Center of Tianjin Medical University (Tianjin, China). All animals were randomly allocated to experimental groups. The care and treatment of the mice were performed in accordance with the Guidelines for Laboratory Animal Care and were approved by the Animal Ethics Committee of Tianjin Medical University (Tianjin, China).

For the tumor formation study, an orthotopic model of pancreatic cancer was established. The mouse abdomens were prepared with betadine solution, and an ~1-cm-wide incision was made in the upper left of each abdomen. We gently grasped the tip of the pancreas and externalized the pancreas and spleen in a lateral direction to completely expose them. A needle was inserted into the pancreas tail and positioned in the pancreatic head region. A quantity of 1 × 10^6^ Panc02 control cells or Panc02 miR-128-overexpressing cells suspended in 50 ml of PBS was then slowly injected using a 27-gauge needle. The spleen was then restored to the appropriate position in the abdomen, and the skin and peritoneum were closed with 3-0 vicryl sutures (*n* = 5/group). Three weeks later, all mice were euthanized, and tumor tissues were collected for further study.

### Flow Cytometry Analysis

The tumors were weighed, minced into small fragments, and digested at 37°C in 10 ml of digestion solution [PBS supplemented with type I Collagenase (200 U/ml), Hyaluronidase, and DNase I (100 μg/ml)] for 60 min. Single-cell suspensions were obtained by grinding the digested tissues and filtering them through a 70-μm cell strainer (BD Biosciences). The immune cells were isolated using Ficoll density gradient centrifugation. Freshly isolated immune cells were stained with antibodies for 30 min at 4°C. The following monoclonal anti-mouse antibodies were used: CD45-PECy5.5 (eBioscience), CD3-pecy7 (eBioscience), CD8-APC (eBioscience), NK1.1-APC (eBioscience), F4/80-APC (eBioscience), CD11b-pecy7 (eBioscience), CD11c-APC (eBioscience), and MHCII-PE (eBioscience). Flow cytometry was performed on a FACS Canto II flow cytometer (BD Biosciences), and the data were analyzed using FlowJo software (TreeStar, Ashland, OR).

### HE Staining and Immunohistochemistry (IHC)

The livers from the tumor-bearing mice were dissected and fixed with 4% paraformaldehyde for 48 h. The liver paraffin sections (5 μm) were stained with hematoxylin and eosin (HE) staining buffer to examine liver metastasis. For immunohistochemical evaluation, the tumor tissues were embedded in paraffin after being fixed in 4% paraformaldehyde for 48 h and then cut into sections of 5 μm in thickness. Paraffin sections were immunostained with antibodies against CD8 (1:100; ABclonal, China), CD11c (1:100; ABclonal, China), CD49b (1:100; ABclonal, China), and F4/80 (1:200; BioLegend, USA) overnight at 4°C. Next, anti-rabbit antibodies (1:200; CST, USA) and DAB solution (OriGene, China) were added. Images were acquired with a microscope (Olympus, Japan).

### *In vitro* Co-cultivation of Tumor Cells With Macrophages or DCs

For co-cultivation with macrophages, bone marrow cells were isolated from the femur and tibia of C57BL/6 mice, then cultured in complete RPMI-1640 supplemented with 10% FBS, 1% penicillin–streptomycin, and 20 ng/ml of recombinant mouse M-CSF (PeproTech, USA) in a CO_2_ incubator for 5 days at 37°C to differentiate into macrophages. For macrophages from the peritoneal cavity, thioglycollate-elicited peritoneal macrophages were collected 96 h after introperitoneal injection (ip) of a 3% thioglycollate solution. Both macrophages (5 × 10^4^ per well) were separately seeded in 24-well-plates for 24 h, incubated in serum-free medium for 2 h, and co-cultivated with 2 × 10^4^ GFP^+^ control or miR-128 overexpression Panc02 cells at 37°C for 4 h. Then, the cells were stained with anti-mouse F4/80-APC (Sungene, China) and analyzed on a FACS Canto II flow cytometer (BD Biosciences). A total of 10,000 cells in each sample were analyzed. Phagocytosis was calculated as the percentage of F4/80^+^GFP^+^ cells among F4/80^+^ macrophages.

For co-cultivation with DCs, we collected bone marrow from mouse femurs and tibiae and cultured it in complete RPMI-1640 supplemented with 10% FBS, 1% penicillin–streptomycin, 20 ng/ml of recombinant mouse GM-CSF (PeproTech, USA), and 10 ng/ml of recombinant mouse IL-4 (PeproTech, USA) in a CO_2_ incubator for 7 days at 37°C to differentiate into DCs. We co-cultivated DCs with control or miR-128-overexpressing Panc02 cells in a ratio of 1:1 for 48 h, and the expression of costimulatory molecules (CD80, CD86) and antigen peptide (MHC-I, MHC-II) on DCs (CD11c^+^) were analyzed by flow cytometry. The antibodies were purchased from eBioscience.

### Bioinformatics Analysis

LinkedOmics (https://www.linkedomics.org) (28) was used to assess the correlation between miR-128 and overall survival of PDAC and the correlation between CD47 and ZEB1. The correlation was evaluated using Spearman's correlation analysis.

### Statistical Analysis

The data are presented as the mean ± SEM. All statistical analyses were performed using GraphPad Prism software unless stated otherwise. Unpaired two-tailed Student's *t*-tests were performed for comparisons. Comparisons among three groups were determined by two-way ANOVA. Differences were considered to be statistically significant at *p* < 0.05.

## Results

### miR-128 Is Related to Overall Survival of PDAC and Inversely Correlated With CD47, Which Is Positively Related to ZEB1 in PDAC

**Figure 1:**
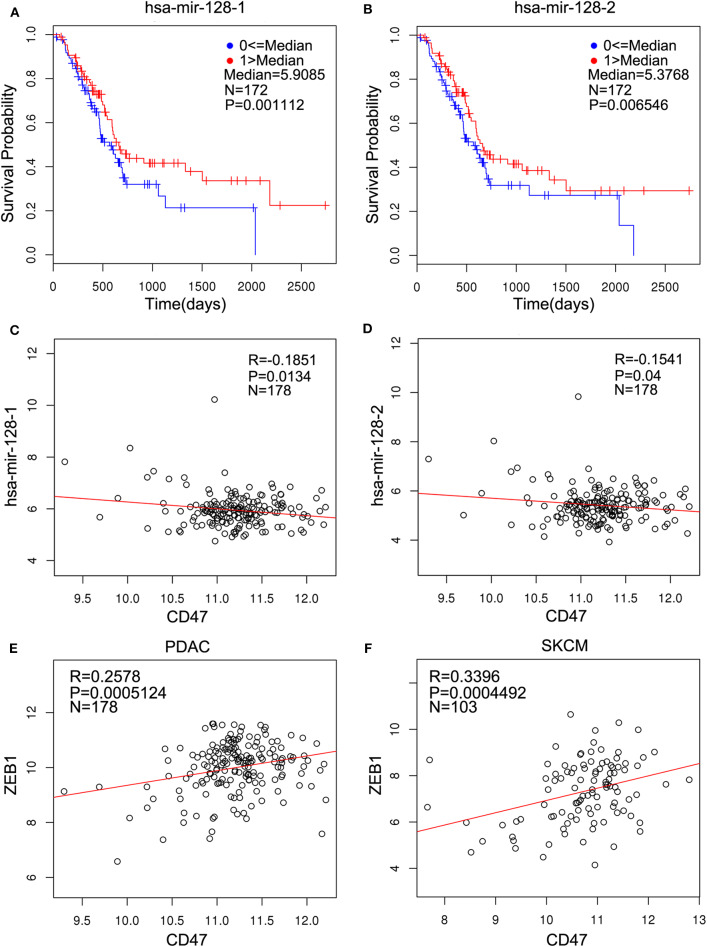
miR-128 is related to overall survival of pancreatic adenocarcinoma (PDAC) and inversely correlated with cluster of differentiation 47 (CD47), which is positively related to zinc finger E-box-binding homeobox 1 (ZEB1) in PDAC. The clinical relevance was analyzed using the LinkedOmics database. **(A,B)** High expression of hsa–mir-128-1 (**A**, *N* = 172, *p* < 0.01) and hsa–mir-128-2 (**B**, *N* = 172, *p* < 0.01) indicates better progression-free survival in patients with PDAC. **(C,D)** Hsa–mir-128-1 (**C**, *N* = 178, *R* = −0.1851, *p* < 0.05) and hsa–mir-128-2 (**D**, *N* = 178, *R* = −0.1541, *p* < 0.05) are inversely related to CD47 in PDAC. **(E,F)** CD47 is positively related to ZEB1 in PDAC **(E**, *N* = 178, *R* = 0.2578, *p* < 0.001) and skin cutaneous melanoma (SKCM) **(F**, *N* = 103, *R* = 0.3396, *p* < 0.001).

miR-128 is remarkably conserved: both human and house mouse have the same mature miR-128 with identical sequences. We analyzed data from the LinkedOmics database and found that high expression levels of both miR-128-1 and miR-128-2 were found to be associated with increased probability of overall survival in 172 PDAC samples (Figures 1A,B). Both miR-128-1 and miR-128-2 were inversely correlated with CD47 in PDAC (Figures 1C,D). In addition, the expression of CD47 was positively correlated with ZEB1 in 178 PDAC and 103 skin cutaneous melanoma (SKCM) patients (Figures 1E,F). This suggests that miR-128 is closely related to the expression of CD47 and ZEB1 in clinical patients who suffer from PDAC.

### Overexpression of miR-128 Inhibited Tumor Growth and Metastasis in PDAC

**Figure 2:**
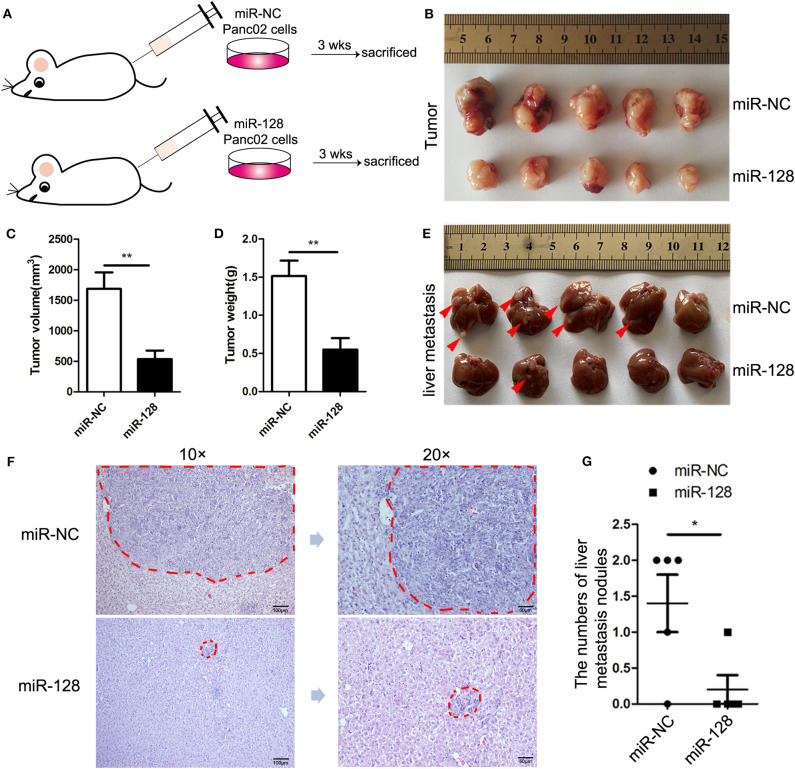
Overexpression of miR-128 inhibited tumor growth and metastasis in PDAC. To establish the orthotopic mouse model of PDAC, miR-NC/miR-128 stable overexpression Panc02 cells were directly injected into the pancreas of C57BL/6 mice (*n* = 5/group). **(A)** A schematic diagram of the mouse model of PDAC. **(B)** Typical tumor photograph. **(C)** Tumor volume. **(D)** Tumor weight. **(E)** Macroscopic image of liver metastasis. The red arrows point to the metastatic sites. **(F)** Hematoxylin and eosin (HE) staining of liver metastasis; the areas of metastasis were circled in red. **(G)** The statistics of liver metastasis. The results represent the mean ± SEM from three independent experiments. Comparisons between groups were determined by Student's *t*-test. *p < 0.05, **p < 0.01.

To determine the impact of miR-128 on PDAC *in vivo*, miR-128, or miR-NC overexpression lentiviruses were synthesized and used to infect Panc02 cells, and miR-128 or miR-NC stable overexpression Panc02 cells were constructed. Before establishing an orthotopic mouse model of PDAC, we detected the overexpression of miR-128 in the relevant Panc02 and miR-NC cells with qRT-PCR (Supplemental Figure 1). Then, we established an orthotopic mouse model of PDAC. MiR-NC/miR-128 stable overexpression Panc02 cells were directly injected into the pancreas of C57BL/6 mice, respectively (Figure 2A). Three weeks later, all the mice were euthanized, and the tumors and livers were acquired. The primary tumor sizes and tumor weights were statistically analyzed. After the livers were treated with formalin fixation and paraffin embedding and cut into serial sections, hematoxylin, and eosin (HE) staining was performed. The results indicated that mice bearing miR-128 stable overexpression Panc02 cells showed reduced tumor size (Figures 2B,C) and tumor weight (Figure 2D) compared with mice bearing miR-NC Panc02 cells. Moreover, the overexpression of miR-128 inhibited the metastasis of *in situ* PDAC to livers (Figure 2E). HE staining of livers from tumor-bearing mice also showed that overexpression of miR-128 inhibited metastasis to livers (Figures 2F,G). These results suggest that miR-128 could inhibit tumor growth and metastasis in PDAC.

### Overexpression of miR-128 Enhanced Anti-tumor Immunity in PDAC

**Figure 3:**
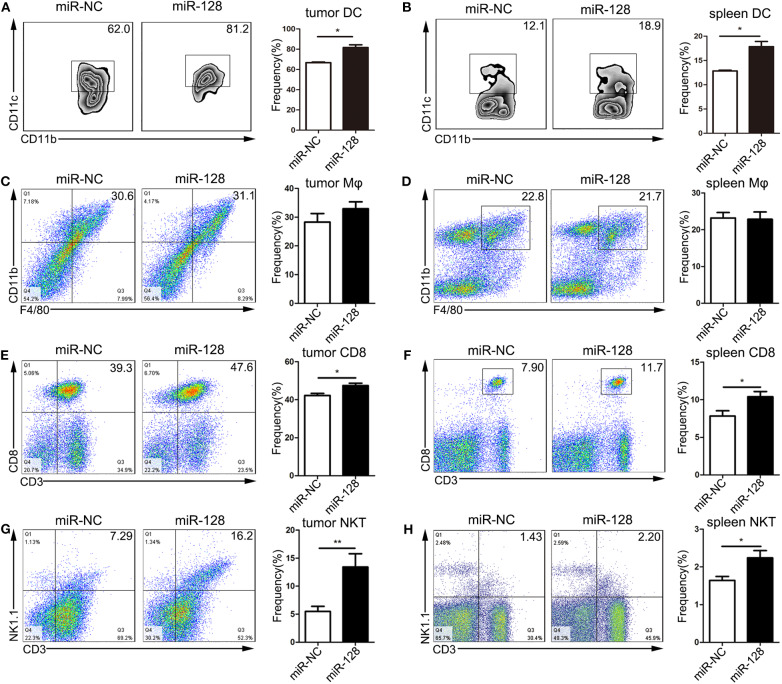
Overexpression of miR-128 enhanced anti-tumor immunity. The tumors and spleens were isolated from *in situ* pancreas-tumor-bearing mice and stained for flow cytometry analysis. The dendritic cells (DCs) **(A,B)**, macrophages **(C,D)**, CD8^+^ T cells **(E,F)**, and natural killer T (NKT) cells **(G,H)** were analyzed by flow cytometry analysis. The results represent the mean ± SEM from three independent experiments. Comparisons between groups were determined by Student's *t*-test. *p < 0.05, **p < 0.01.

To determine whether overexpression of miR-128 could enhance anti-tumor immunity, tumors, and spleens from the orthotopic mouse model of PDAC were isolated and stained for flow cytometry analysis. The percentages of DCs were significantly increased in the tumors (Figure 3A) and spleens (Figure 3B) of the miR-128 overexpression group compared with those of the miR-NC group. The gating strategies for DCs are shown in Supplemental Figures 2, 3. However, there was no difference in the percentage of macrophages in the tumors (Figure 3C) or spleens (Figure 3D) of the two groups. In addition, CD8^+^ T lymphocytes were significantly upregulated in the tumors (Figure 3E) and spleens (Figure 3F) of miR-128 overexpression group. The percentage of natural killer T (NKT) cells was also increased in both tumors (Figure 3G) and spleens (Figure 3H) of the miR-128 overexpression group. Moreover, we also analyzed the liver metastases microenvironment from the orthotopic mouse model of PDAC. Immunohistochemical staining of sections of tumor-bearing mouse livers revealed that compared with the miR-NC group, the group with overexpression of miR-128 had increased infiltration of CD8^+^ T cells, DCs (CD11C^+^), macrophages (F4/80^+^), and NKT cells (CD49b^+^) in the livers (Supplemental Figure 4). Overall, overexpression of miR-128 could enhance anti-tumor immunity in PDAC.

### miR-128 Overexpression on Panc02 Cells Enhanced the Phagocytosis of Macrophages and the Activity of DCs

**Figure 4:**
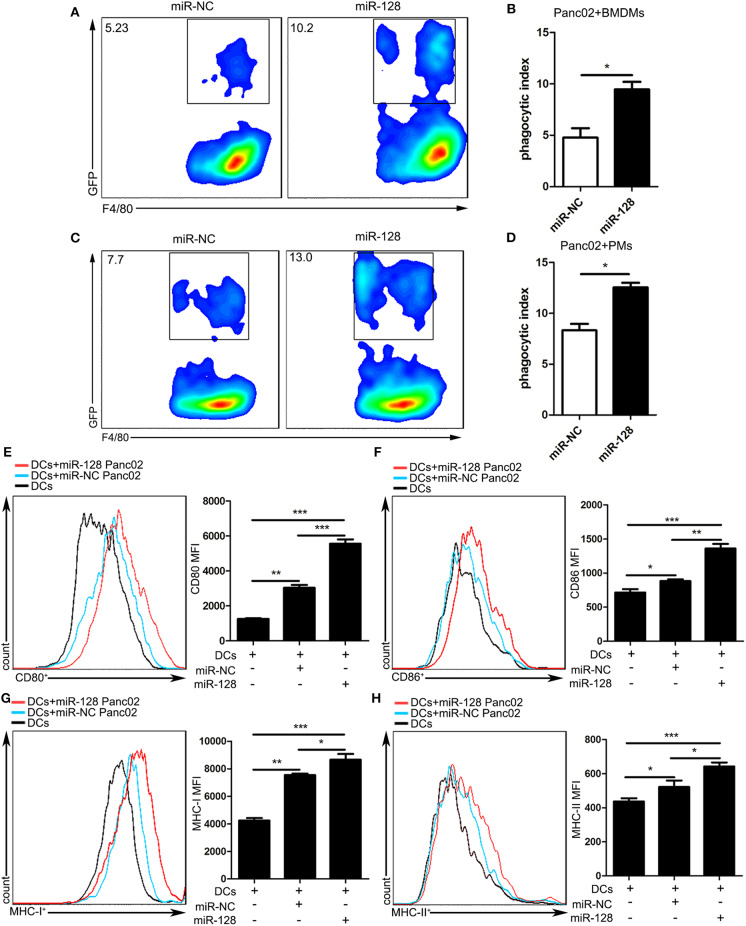
Overexpression of miR-128 in Panc02 cells enhanced the phagocytosis of macrophages and the activity of DCs. miR-NC or miR-128 overexpression Panc02 cells were incubated with mouse bone-marrow-derived macrophages (BMDMs) or peritoneal-cavity-derived macrophages for 4 h, stained with F4/80-APC antibody, and analyzed by flow cytometry. **(A,C)** Representative plots show the percentage of F4/80^+^GFP^+^ macrophages phagocytosing cancer cells among F4/80^+^ BMDMs and peritoneal-cavity-derived macrophages. **(B,D)** Statistical analysis of the phagocytosis of cancer cells by both BMDMs and peritoneal-cavity-derived macrophages. MiR-NC or miR-128 overexpression Panc02 cells were incubated with mouse bone-marrow-derived dendritic cells (BMDCs) for 48 h. The expression of costimulatory molecules CD80 **(E)** and CD86 **(F)** and antigen peptides MHC-I **(G)** and MHC-II **(H)** on DCs were analyzed by flow cytometry. These results represent the mean ± SEM from three independent experiments. Comparisons among three groups were determined by ANOVA. *p < 0.05, **p < 0.01, and ***p < 0.001.

As CD47-mediated SIRPa signals play an important role in the phagocytosis of numerous hematologic and solid cancers by macrophages, we attempted to explore the function of miR-128 in Panc02 cells to be phagocytosed by macrophages. First, we isolated macrophages from mouse bone marrow (BMDMs), co-cultivated them with miR-NC, or miR-128 stable overexpression Panc02 cells for 4 h, then harvested and stained them with anti-mouse F4/80-APC for flow cytometry. Compared with miR-NC Panc02 cells, miR-128 overexpression Panc02 cells enhanced phagocytosis by macrophages (Figures 4A,B). We also performed *in vitro* phagocytosis assays by incubating peritoneal-cavity-derived macrophages (PMs) with miR-NC or miR-128 stable overexpression Panc02 cells and measuring the phagocytosis. As expected, we found that forced expression of miR-128 increased the phagocytosis index compared with the control group (Figures 4C,D). These results indicate that miR-128 might serve as a potent therapeutic target against PDAC on account of its close relationship with macrophage phagocytosis.

Previous studies showed that CD47 blockade in tumor models requires DCs to activate adaptive immune responses. To explore the impact of miR-128-overexpressing Panc02 cells on the activity of DCs, we cultured murine bone marrow (BM) cells with granulocyte macrophage colony-stimulating factor (GM-CSF) to generate bone-marrow-derived dendritic cells (BMDCs) and detected the phenotype of BMDCs by flow cytometry at 6 days (Supplemental Figure 5). Then, we co-cultivated BMDCs with miR-NC or miR-128 stable overexpression Panc02 cells. The flow cytometry analysis showed that miR-128-overexpressing Panc02 cells could enhance the expression of costimulatory molecules (CD80, CD86) and antigen peptides (MHC-I, MHC-II) on DCs (Figures 4E–H), which indicates that miR-128-overexpressing Panc02 cells could enhance the antigen presentation and immune activation ability of DCs.

### miR-128 Inhibited the Cell Proliferation, Migration, and Invasion of PDAC Cells

**Figure 5:**
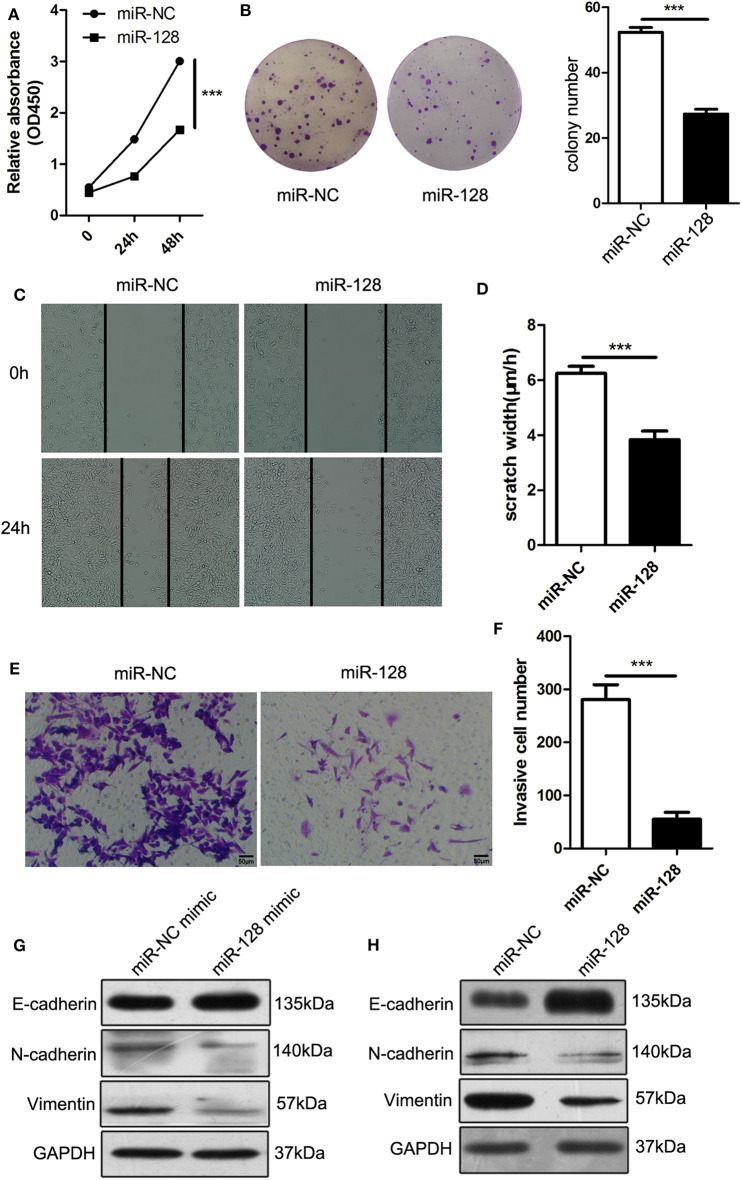
MiR-128 inhibited the cell proliferation, migration, and invasion of PDAC cells. **(A)** Cell Counting Kit-8 (CCK-8) assay: the proliferation of miR-128 lentivirus-infected cells was lower than that of the control group at 24 and 48 h. **(B)** Clonogenicity assays: the miR-128 lentivirus-infected cells had a significantly lower survival rate than the control cells. **(C,D)** Wound healing assay: the migration of miR-128 lentivirus-infected cells was slower than that of the control cells. **(E,F)** Transwell assay: the percentage of cells that invaded through the Matrigel was lower in miR-128 lentivirus-infected cells than in the control cells. **(G,H)** The expression of genes associated with epithelial–mesenchymal transition (EMT) after mimic transfection and lentivirus infection. The results represent the mean ± SEM from three independent experiments. Comparisons between groups were determined by Student's *t*-test. ***p < 0.001.

To explore the effects of miR-128 on the biological function of PDAC, we first explored cell proliferation via Cell Counting Kit-8 (CCK-8) assays and clonogenicity assays; the results showed that overexpression of miR-128 decreased cell proliferation (Figure 5A) and clonogenicity of Panc02 cells (Figure 5B). We also performed a wound healing assay and invasion assay; the results revealed that the percentages of migration (Figures 5C,D) and invasion (Figures 5E,F) of miR-128-overexpressing Panc02 cells were significantly lower than those of miR-NC cells. Furthermore, we assessed the expression of genes that were known to be associated with metastasis. E-cadherin was upregulated, while N-cadherin and Vimentin were downregulated in miR-128 mimic-transfected cells (Figure 5G) and miR-128 stable overexpression Panc02 cells (Figure 5H). These results indicate that miR-128 could inhibit the migration and invasion of Panc02 cells and regulate the progress of EMT.

### miR-128 Promotes Anti-tumor Immunity in PDAC Through Regulating the ZEB1/CD47 Axis

**Figure 6:**
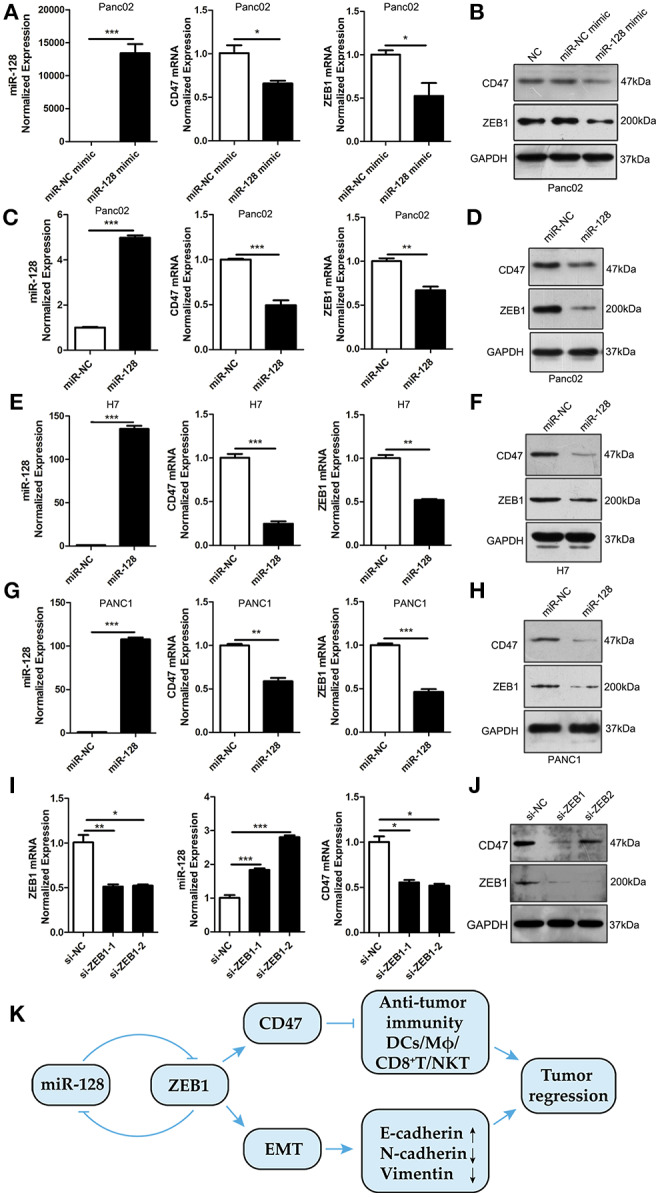
miR-128 promotes anti-tumor immunity through the ZEB1/CD47 axis. **(A,B)** The expression of CD47 and ZEB1 were downregulated in miR-128 mimic-transfected Panc02 cells. **(C,D)** The expression of CD47 and ZEB1 were downregulated in miR-128 lentivirus-infected Panc02 cells. **(E,F)** The expression of CD47 and ZEB1 were downregulated in miR-128 mimic-transfected H7 cells. **(G,H)** The expression of CD47 and ZEB1 were downregulated in miR-128 mimic-transfected PANC1 cells. **(I,J)** The expression of ZEB1, miR-128, and CD47 in ZEB1 knockdown Panc02 cells. **(K)** A schematic of the molecular mechanism by which miR-128 causes tumor regression. These results represent the mean ± SEM from three independent experiments. Comparisons between groups were determined by Student's *t*-test. *p < 0.05, **p < 0.01, and ***p < 0.001.

To explore the molecular mechanism by which miR-128 regulates anti-tumor immunity, we detected the regulation of miR-128 to ZEB1. The expression of miR-128 in miR-128 mimic-transfected cells was validated, and the results by qRT-PCR and Western blot showed that miR-128 mimic-transfected Panc02 cells decreased the expression of CD47 and ZEB1 at both mRNA levels and protein levels (Figures 6A,B). Consistent with the results attained by miR-128 or miR-NC mimic transfection, the expression of miR-128 in miR-128 lentivirus-infected cells was increased; the expressions of CD47 and ZEB1 were decreased at both mRNA levels and protein levels (Figures 6C,D). Moreover, the miR-128 mimic-transfected H7 or PANC1 cells decreased the expression of CD47 and ZEB1 at both mRNA levels and protein levels (Figures 6E–H). When ZEB1 was knocked down in Panc02 cells, miR-128 was upregulated, while CD47 was downregulated (Figures 6I,J). Taken together, miR-128 is a crucial modulator of ZEB1, and regulated by ZEB1 in turn, it further inhibits the expression of CD47. This enhanced the anti-tumor immune response while regulating EMT through ZEB1, inhibiting tumor growth and metastasis, and ultimately causing tumor regression (Figure 6K).

## Discussion

Numerous studies have shown that microRNAs are clinically related to tumor proliferation, invasion, metastasis, and chemo-resistance in patients suffering from PDAC (29–31). In recent years, inhibitory immune checkpoint blockade has been one of the most significant advances in anti-tumor therapy (32, 33), while progress has been made in clarifying the roles of miRNAs as modulators of immune checkpoint molecules in regulating immune responses and their potential as targets for cancer therapy (34, 35).

Accumulating evidence suggests that the blockade of CD47 and its ligands signal regulatory protein α (SIRPα) are promising in tumor immunotherapy (10). A number of agents [such as B6H12 (36), Hu5F9-G4 (37), etc.] targeting either CD47 or SIRPα have showed efficacy against tumors, and several agents are being tested in phase I clinical trials in progress (11). ZEB1 was shown to be important for tumorigenicity and metastasis (38, 39). It triggers a microRNA-mediated feedforward loop that stabilizes EMT and promotes invasion of cancer cells (40) and could target CD47 (18). Studies have reported that miR-128 could modulate chemosensitivity and EMT in prostate cancer and esophageal squamous cell cancer (41, 42); however, little is known about the regulation of miR-128 in PDAC. In this study, we found that high expression of miR-128 is associated with increased probability of overall survival and inversely correlated with CD47 in PDAC. The expression of CD47 was positively correlated with ZEB1 both in PDAC and in SKCM patients. Furthermore, functional studies showed that the restoration of miR-128 in Panc02 cells attenuated cell proliferation, migration, and invasion, inhibited the progression of EMT, and increased the expression of E-cadherin, while downregulating N-cadherin and Vimentin. miR-128 could regulate ZEB1 and can be regulated by ZEB1, further impacting CD47 and EMT. *In vivo* experiments showed that overexpression of miR-128 could significantly inhibit tumor growth and metastasis in PDAC.

CD47 is a promising strategy for cancer treatment based on the modulation of both innate and adaptive immune responses to tumor cells. One mechanism behind CD47-mediated immune evasion is that it can interact with SIRPα on myeloid cells such as macrophages, then transmit a “don't eat me” signal to prevent cell engulfment (9, 11). Another study reported that the therapeutic efficacy of CD47 blockade in tumor models requires DCs to activate adaptive immune responses (12); DCs, but not macrophages, sense tumor mitochondrial DNA for cross-priming through the CD47–SIRPα signal (43). Moreover, adaptive immune response, especially that mediated by CD8^+^ T cells, plays a critical role in anti-CD47-blockade-induced tumor inhibition (12). NKT cells are distinct population of T cells with the characteristics of both innate and adaptive immunity (44). Of the total NKT cells, 80% are type I NKT cells, which are defined as invariant NKT (iNKT) (45); they can produce interferon-gamma (IFN-γ) to activate NK and CD8^+^ T cells and by activating DCs to make interleukin-12 (IL-12) (44). In turn, the iNKT cells can be stimulated by IL-12 released from DCs or macrophages through IL-12 receptors expressed on iNKT cells (46, 47). As CD47 is downstream of ZEB1 (18), it might be a critical link between miR-128 and anti-tumor immunity. In the current study, we analyzed the immune microenvironment and demonstrated that the percentage of DCs was significantly increased in miR-128 overexpression tumor-bearing mice, along with increased CD8^+^ T cells and NKT cells, leading to the enhancement of anti-tumor immunity. Therefore, miR-128 regulated the infiltration of anti-tumor immune cells, including DCs, CD8^+^ T cells, and NKT cells, in the immune microenvironment through the ZEB1/CD47 axis, accompanied by the inhibition of the EMT process through ZEB1, ultimately inhibiting tumor growth and metastasis. Meanwhile, we co-cultivated miR-NC or miR-128 stable overexpression Panc02 cells with macrophages or DCs, and we found that miR-128-overexpressing Panc02 cells enhanced the phagocytosis of macrophages and the activity of DCs. A more in-depth mechanism of critical anti-tumor immune cells interacting with cancer cells through CD47 needs to be further studied.

Overall, miR-128 could serve as an important regulator of tumor immunity through the ZEB1/CD47 axis and EMT in PDAC. These results provide a rationale for an innovative preclinical combination immunotherapy based on CD47 regulation along with EMT inhibitors in patients with highly aggressive and metastatic PDAC.

## Data Availability Statement

The datasets generated for this study are available on request to the corresponding author.

## Ethics Statement

The animal study was reviewed and approved by Animal Ethics Committee of Tianjin Medical University.

## Author Contributions

QX and RZ conceived and designed the experiments. QX, YC, and G-ZY performed the majority of experiments, with the help of J-YZhan, L-JZ, X-DG, J-YZhao, Z-YX, and YL. QX, YC, and RZ analyzed the data. QX and RZ wrote the paper.

## Conflict of Interest

The authors declare that the research was conducted in the absence of any commercial or financial relationships that could be construed as a potential conflict of interest.
