# Supplementary material for: miR-128 Regulates Tumor Cell CD47 Expression and Promotes Anti-tumor Immunity in Pancreatic Cancer
Source: Front Immunol. 2020 May 27;11:890. doi: 10.3389/fimmu.2020.00890 (PMC7267029; doi:10.3389/fimmu.2020.00890)
Supplement: Supplementary file 1 [file Data_Sheet_1.docx]

**Supplemental Figures and Legends**

**
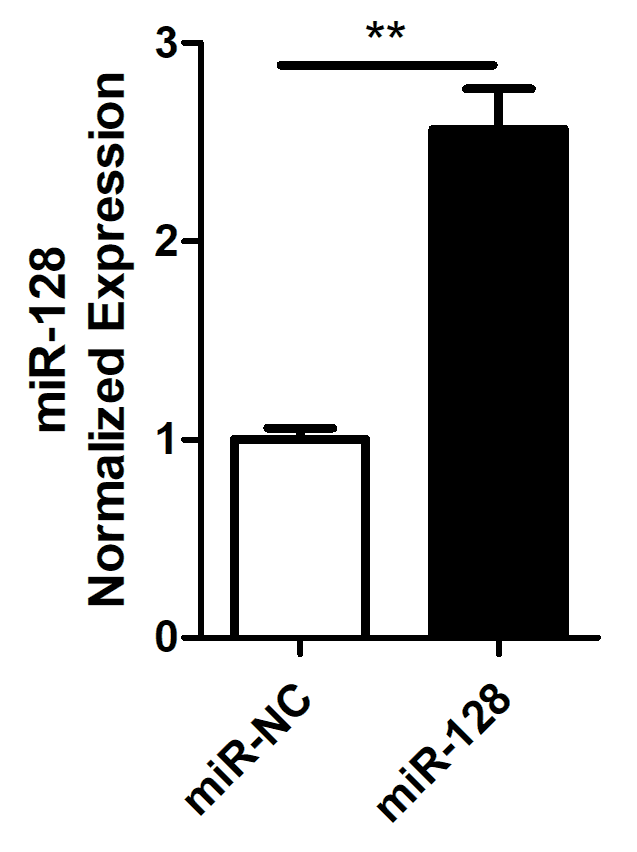
**

**Supplemental Figure 1: The expression of miR-128 in miR-128 lentivirus- infected cells.** The normalized expression of miR-128 was detected by qRT-PCR in miR-NC and miR-128 lentivirus-infected Panc02 cells. The result represents mean ± SEM from three independent experiments. Comparisons between two groups were determined by Student’s t-tests (**p < 0.01).


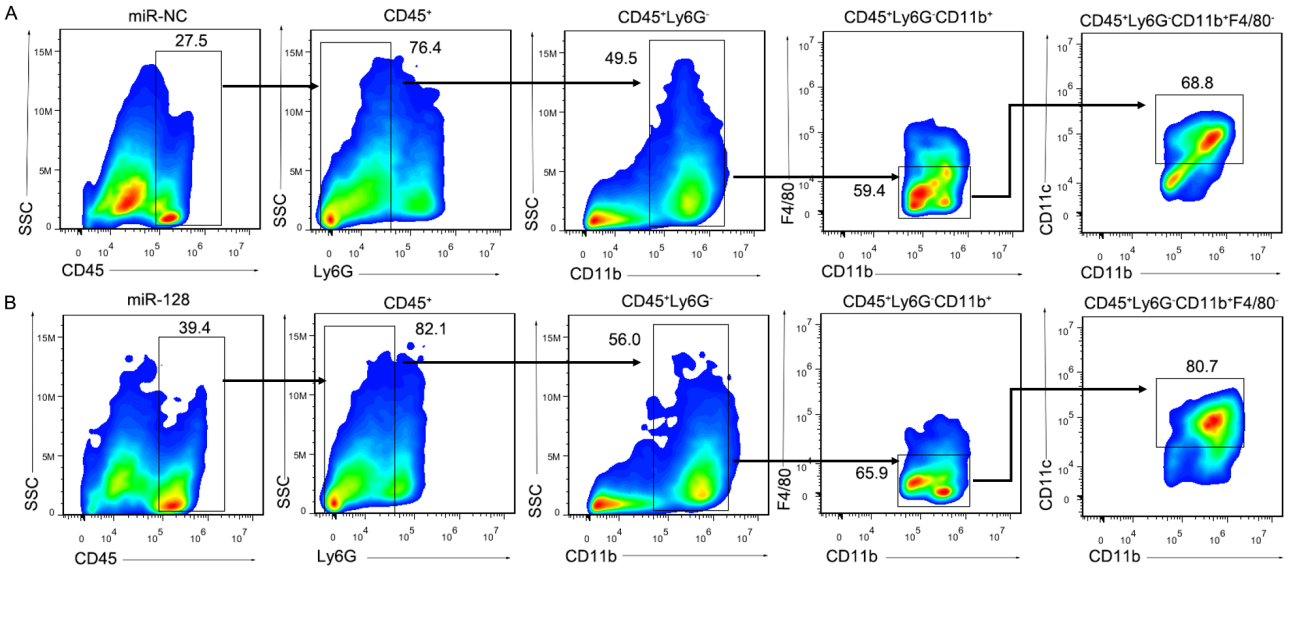


**Supplemental Figure 2：Overexpression of miR-128 enhanced the percentage of DC cells in tumor.** Single tumor cell suspension from the orthotopic mouse model of PDAC was stained with different fluorophore-conjugated antibodies and analyzed by flow cytometry. The CD45^+^ cells were selected for further analysis to identify DC cells (Ly6G^-^CD11b^+^F4/80^-^CD11c^+^) in tumors. The graph shows detailed characterization process of DC cells in tumors between miR-NC **(A)** and miR-128 **(B)** overexpression tumor-bearing mice.

**
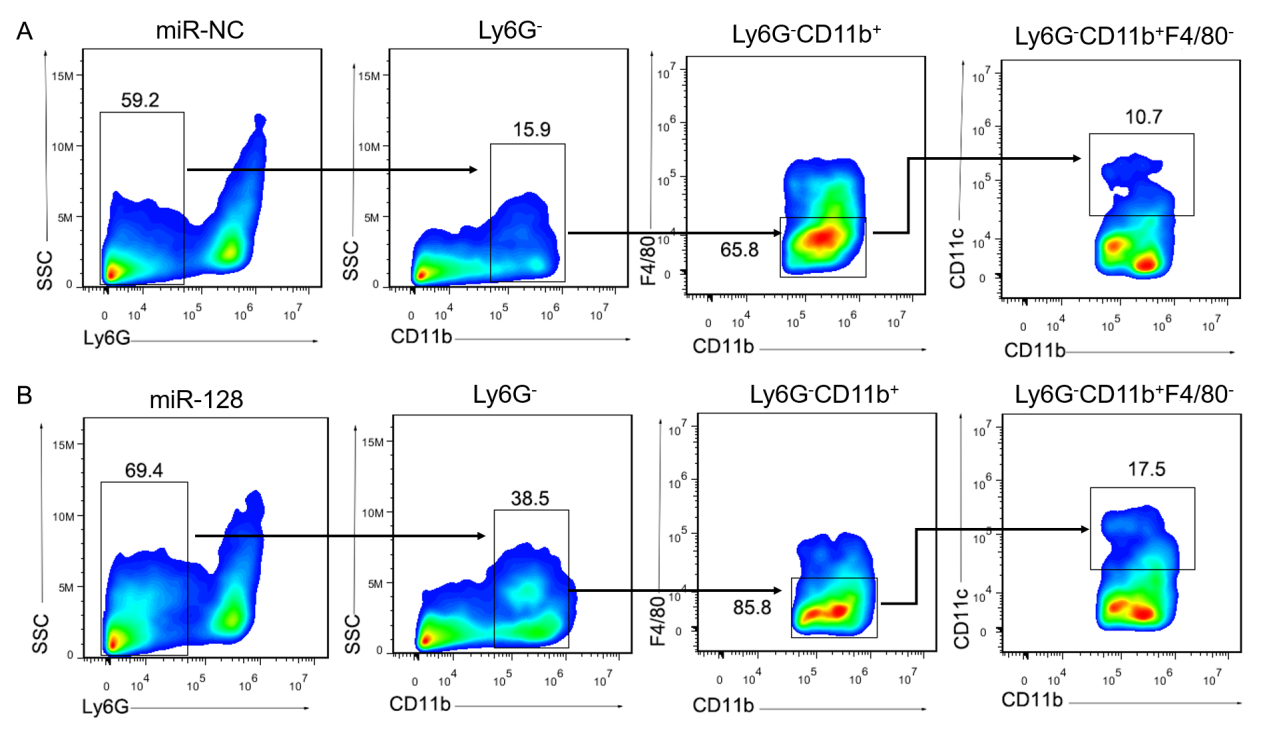
**

**Supplemental Figure 3: Overexpression of miR-128 enhanced the percentage of DC cells in spleen.** Single cell suspension from spleen of *in situ* pancreas-tumor-bearing mice was stained for flow cytometry analysis. DC cells (Ly6G^-^CD11b^+^F4/80^-^CD11c^+^) in spleen were analyzed. Detailed characterization process of DC cells in spleen was showed between miR-NC **(A)** and miR-128 **(B)** overexpression tumor-bearing mice.

**
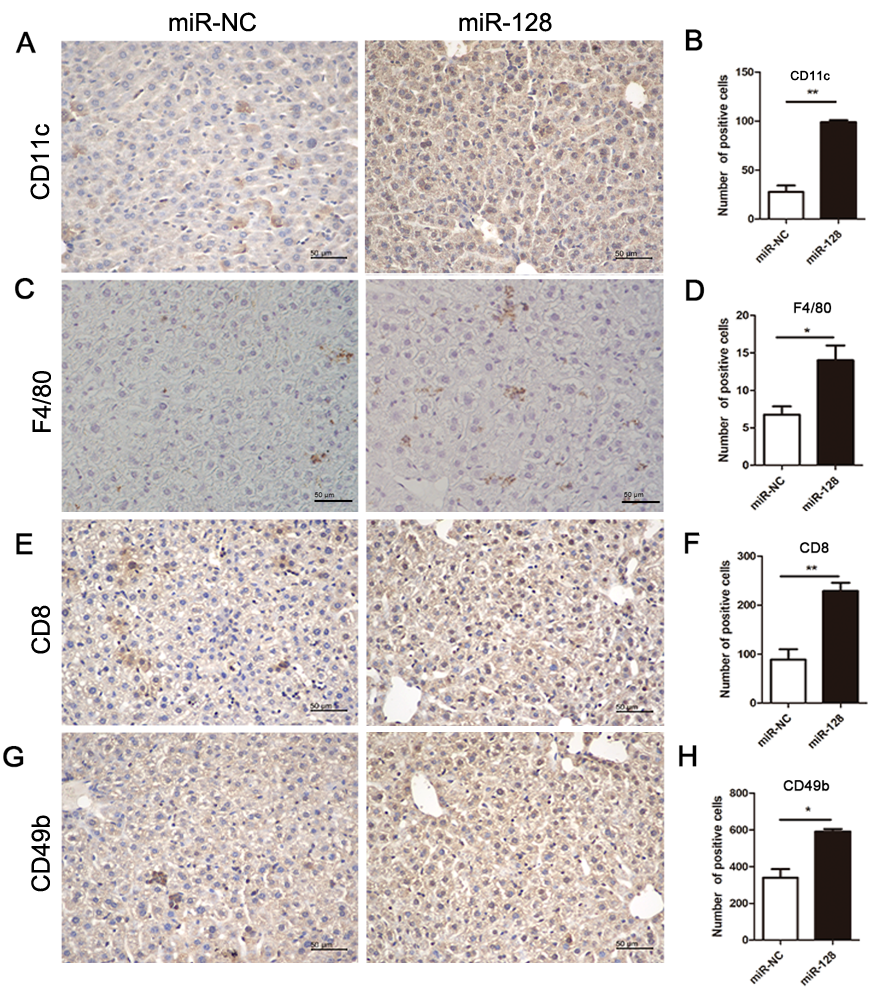
**

**Supplemental Figure 4: Overexpression of miR-128 enhanced the infiltration of immune cells in liver.** Immune cells in liver microenvironment from the orthotopic mouse model of PDAC were measured by IHC **(A, C, E, G)**. (**A)** CD11c^+^ DC cells; (**C)** F4/80^+^ macrophages; **(E)** CD8^+^T cells; **(G)** CD49b^+^ NKT cells. Statistical analysis of immune cells in liver between miR-NC and miR-128 overexpression tumor-bearing mice **(B, D, F, H)**. Comparisons between groups were determined by Student’s t test (* p < 0.05, ** p < 0.01).


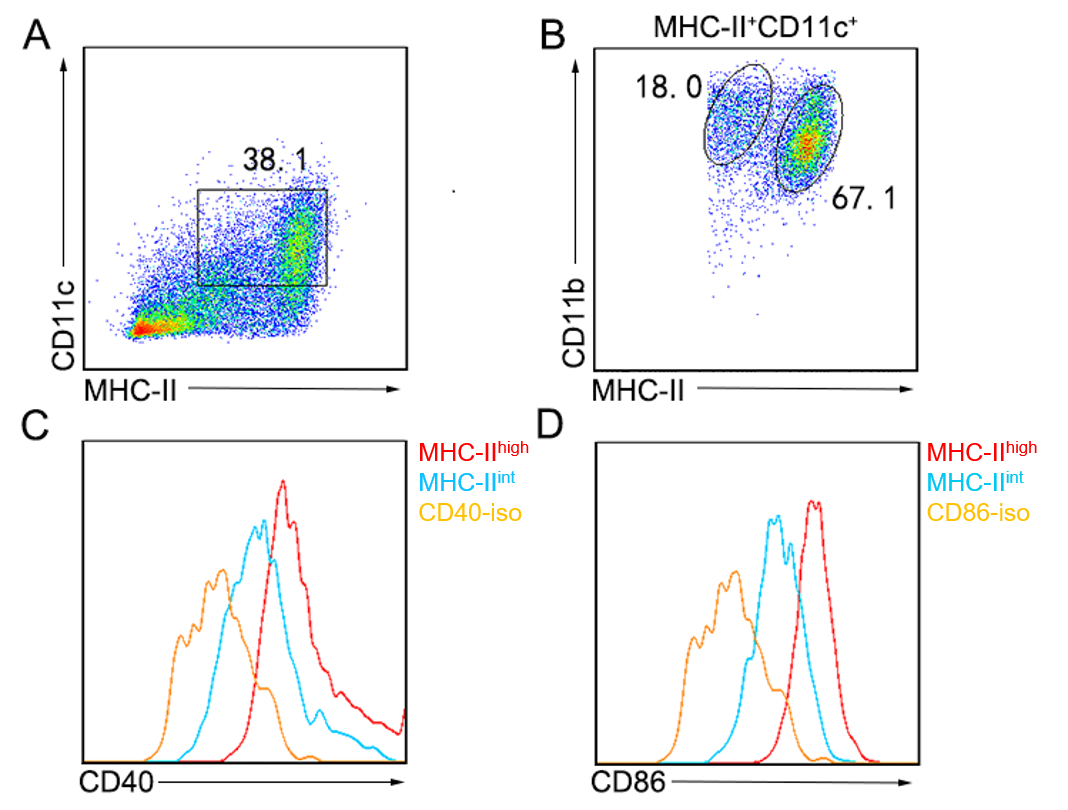


**Supplemental Figure 5: Induction of BMDC maturation *in vitro*.** Culture of murine bone marrow cells with GM-CSF to generate BMDCs and detection the phenotype of BMDCs by flow cytometry at 6 days. **(A)** Representative gates of MHC-II^+^CD11c^+^ cells; **(B)** Representative gates of MHC-II^int^CD11b^hi^(left) and MHC-II^hi^CD11b^lo^(right); The expression of CD40 **(C)** and CD86 **(D)** in MHC-II^hi^CD11b^lo^(red), MHC-II^int^CD11b^hi^(blue) and isotype control (yellow).
